# Hepatoprotective effects of *Micromeria croatica* ethanolic extract against CCl_4_–induced liver injury in mice

**DOI:** 10.1186/s12906-015-0763-8

**Published:** 2015-07-15

**Authors:** Sanda Vladimir-Knežević, Olga Cvijanović, Biljana Blažeković, Marija Kindl, Maja Bival Štefan, Robert Domitrović

**Affiliations:** Department of Pharmacognosy, Faculty of Pharmacy and Biochemistry, University of Zagreb, Zagreb, Croatia; Department of Anatomy, School of Medicine, University of Rijeka, Rijeka, Croatia; Department of Chemistry and Biochemistry, School of Medicine, University of Rijeka, B. Branchetta 20, 51000 Rijeka, Croatia

**Keywords:** *Micromeria croatica*, Hepatoprotection, Oxidative stress, Inflammation, Liver fibrosis

## Abstract

**Background:**

*Micromeria croatica* (Pers.) Schott is an aromatic plant from Lamiaceae family previously found to possess potent *in vitro* antioxidant activity which is mainly attributed to the high level of polyphenolic substances. The aim of this study was to investigate the hepatoprotective activity and possible underlying mechanisms of *Micromeria croatica* ethanolic extract (MC) using a model of carbon tetrachloride (CCl_4_)-induced liver injury in mice.

**Methods:**

Male BALB/cN mice were randomly divided into seven groups: control group received saline, MC group received ethanolic extract of *M. croatica* in 5 % DMSO (100 mg/kg b.w., p.o.), and CCl_4_ group was administered CCl_4_ dissolved in corn oil (2 mL/kg, 10 % v/v, *ip*). MC50, MC200 and MC400 groups were treated with MC orally at doses of 50, 200 and 400 mg/kg once daily for 2 consecutive days, respectively, 6 h after CCl_4_ intoxication. The reference group received silymarin at dose of 400 mg/kg. At the end of experiment, blood and liver samples were collected for biochemical, histopathological, immunohistochemical and Western blot analyses. In addition, major phenolic compounds in MC were quantified by HPLC-DAD.

**Results:**

CCl_4_ intoxication resulted in liver cells damage and oxidative stress and triggered inflammatory response in mice livers. MC treatment decreased ALT activity and prevented liver necrosis. Improved hepatic antioxidant status was evident by increased Cu/Zn SOD activity and decreased 4-hydroxynonenal (4-HNE) formation in the livers. Concomitantly, nuclear factor erythroid 2-related factor 2 (Nrf2) and heme oxygenase-1 (HO-1) were overexpressed. The hepatoprotective activity of MC was accompanied by the increase in nuclear factor-kappaB (NF-κB) activation and tumor necrosis factor-alpha (TNF-α) expression, indicating amelioration of hepatic inflammation. Additionally, MC prevented tumor growth factor-β1 (TGF-β1) and α-smooth muscle actin (α-SMA) expression, suggesting the potential for suppression of hepatic fibrogenesis.

**Conclusion:**

These results of the present study demonstrated that MC possesses *in vivo* antioxidant and anti-inflammatory activity and exhibits antifibrotic potential, which are comparable to those of standard hepatoprotective compound silymarin.

## Background

Liver diseases represent one of a major health burdens worldwide and a common cause of death in Western countries [1]. Etiologically, various infectious agents including viruses and hepatotoxic chemicals along with environmental pollutants are responsible for different types of liver disease. Hepatic damage is a fundamental pathological process in most of them, and long-standing liver injury leads to fibrosis, cirrhosis and even hepatocellular carcinoma. Recent research in the field of free radical biology suggested an important pathophysiological role of free radicals and oxidative stress in the development and progression of liver diseases [2]. The overproduction of free radicals and other reactive oxygen species (ROS) in the liver cells is considered to cause oxidative damage of vital cellular macromolecules such as lipids, proteins and nucleic acids, leading to cell dysfunction and death. Natural antioxidants can prevent free radical-mediated oxidative damage to cellular components by acting at different levels in the pathophysiological chain. A number of plant extracts and their constituents have been shown to possess hepatoprotective properties by improving the antioxidant status *in vivo* [3–5]. Plant-derived antioxidants may therefore be particularly important in reducing the incidence of various liver disorders as well as other oxidative stress-related diseases.

In spite of an increasing need for agents to protect the liver from damage, modern medicine still lacks a reliable liver-protective drug. Therefore, numerous edible and medicinal plants have been studied to evaluate their hepatoprotective efficiency. The genus *Micromeria* Bentham (Lamiaceae, Nepetoidae) contains about 70 species with a distribution range extending from the Himalayan region to the Macaronesian Archipelago and from the Mediterranean to South Africa and Madagascar. All species are perennial of annual herbs, subshrub or shrub, often with an aromatic scent [6]. Some of them are traditionally used against colds, headache, stomach diseases, liver and heart disorders, wounds and skin infections, as well as for insecticidal, herbicidal and food-related purposes. Besides being used as culinary herbs, the tea from their leaves has been consumed as a beverage [7–9]. *Micromeria* species are reported to have various *in vitro* biological effects including antioxidant [10], antimicrobial [11], anticholinesterase [12], anti-inflammatory and analgesic activities [13], and therefore they have a great potential for use as herbal drugs or dietary supplements.

*Micromeria croatica* (Pers.) Schott is one of nine *Micromeria* species growing in Croatia. It is an endemic species of Croatia and some neighbouring countries which grows in crevices of calcareous rocks at altitudes of 150 to 2000 m [14]. Previous phytochemical investigations of this plant revealed the presence of flavonoid glycosides of luteolin and apigenin [15] and essential oil rich in caryophyllene oxide [16]. Recently we reported that some *Micromeria* species possess considerable antioxidant activities, mainly attributed to the high level of polyphenolic substances [10]. These results suggested that *Micromeria* species, especially *M. croatica*, could be useful in prevention and/or treatment of human diseases in which free radicals and other ROS have been implicated. Therefore, the present study aimed to evaluate protective effect of *M. croatica* ethanolic extract (MC) against acute liver injury and fibrosis induced by CCl_4_ as well as to elucidate a possible mechanism of its hepatoprotection.

## Methods

### Plant material and extraction

Aerial parts of wild-growing *Micromeria croatica* were collected at the full flowering stage in July 2011 in Croatia from South Velebit Mt. (950 m a.s.l.). The plant sample was authenticated by the Department of Pharmacognosy, Faculty of Pharmacy and Biochemistry and Department of Botany and Botanical Garden, Faculty of Science (University of Zagreb, Croatia) where the voucher specimen has been deposited under the genus number 812a.

Air-dried and pulverized plant material (20.00 g) was extracted with 70 % ethanol (200 mL) using an ultrasonic bath (Bandelin Sonorex Digital 10 P, Berlin, Germany) for 30 min. The extract was then filtered through Whatman No. 1 paper using a Büchner funnel and the residue was then re-extracted with the same solvent (200 mL) as described above. Obtained extracts were combined and lyophilized.

For HPLC analysis, the lyophilized *M. croatica* extract was redissolved in HPLC grade methanol to make 5 mg/mL sample solution, then filtered through a 0.45 μm membrane filter (Millipore, USA). Clear pale green filtrate was obtained and immediately used.

### Chemicals and antibodies

Silymarin, bovine serum albumin (BSA), bovine Cu/Zn superoxide dismutase (SOD), xanthine, xanthine oxidase, cytochrome c and Entelan were purchased from Sigma-Aldrich (St. Louis, MO, USA). Carbon tetrachloride (CCl_4_) was obtained from Kemika, Zagreb, Croatia. Diagnostic kit for the serum alanine aminotransferase (ALT) was from Dijagnostika (Sisak, Croatia). Mouse monoclonal antibodies to tumor necrosis factor-α (TNF-α) (ab1793), α-smooth muscle actin (α-SMA) (ab18460), rabbit polyclonal antibodies to 4-hydroxynonenal (4-HNE) (ab46545), nuclear factor-kappaB (NF-κB), nuclear factor erythroid 2-related factor 2 (Nrf2) (ab31163), heme oxygenase-1 (HO-1) (ab13243) and transforming growth factor-beta1 (TGF-β1) were purchased from Abcam (Cambridge, UK). DAKO EnVision + System was from DAKO Corporation (Carpinteria, CA, USA). Radioimmunoprecipitation assay (RIPA) buffer (sc-24948) and milk blocking reagent were purchased from Santa Cruz Biotechnology (Santa Cruz, CA, USA). Polyvinylidene fluoride (PVDF) membrane was supplied from Roche Diagnostics GmbH (Mannheim, Germany), while peroxidase-labeled goat anti-mouse F(ab’)2 and Amersham ECL Prime from Amersham Pharmacia Biotech (Uppsala, Sweden). All other chemicals were of the highest analytical grade available.

### Determination of phenolic content of *M. croatica*

Identification and quantification of the major phenolic constituents of *M. croatica* ethanolic extract was performed using a slightly modified method of Fecka and Turek [17]. HPLC analysis was conducted on an Agilent 1100 Series instrument (Agilent Technologies, Santa Clara, CA, USA) equipped with an Agilent auto sampler, a quaternary pump, a column thermostat, and a photodiode array detector. The separation was achieved using a LichroCART®250-4 column (250 mm × 4.0 mm i.d.) packed with LiChrospher® 100 RP-18e (5 μm) and protected by a suitable guard column. The chromatographic method used was a gradient elution, using acidified acetonitrile (5 % formic acid in acetonitrile, solvent A) and acidified water (5 % formic acid in water, solvent B), at a flow rate of 0.9 mL/min. The gradient employed was as follows: 0 min, 15 % A; 25 min, 35 % A; 27 min, 70 % A; 32 min, 70 % A; 33 min, 100 % A; 38 min, 100 % A. Solvent solutions were vacuum degassed with ultrasonication prior to usage. The injection volume for the sample was 20 μL. All chromatographic experiments were performed at 20 °C. The polyphenolic profiles were recorded at a wavelength of 330 nm. Chromatographic data were acquired and processed using HP ChemStation software. Phenolic acids were identified in the sample based on their chromatographic retention times and UV spectra, and quantified by comparing integrated peak areas to calibration curves prepared with corresponding analytical standards. The results were expressed in mg per g of the dry extract.

### Animals

Male BALB/cN mice, 2 months old, weight 20-24 g, were obtained from the breeding colony of the School of Medicine, University of Rijeka, Croatia. The animals were housed in standard environmental conditions and had free access to tap water and a standard rodent diet (pellet, type 4RF21 GLP, Mucedola, Italy). All experimental procedures were conducted in compliance with the Declaration of Helsinki principles and approved by the Ethical Committee of the School of Medicine, University of Rijeka, Croatia.

### Experimental design

The experiment mice were randomly divided into seven groups of five animals each. Control group received saline and MC group received ethanolic extract of *M. croatica* dissolved in DMSO and diluted with water (5 % v/v), once daily for 2 days at dose of 400 mg/kg. CCl_4_ group was administered CCl_4_ dissolved in corn oil (2 mL/kg, 10 % v/v), intraperitoneally (*ip*). MC50, MC200 and MC400 groups were treated with MC orally at doses of 50, 200 and 400 mg/kg once daily for 2 consecutive days, respectively, 6 h after CCl_4_ intoxication. The doses used were selected on the basis of our preliminary studies (data not shown). Mice tolerated MC well, without any signs of discomfort. The last group received silymarin at dose of 400 mg/kg, which served as a reference. MC, silymarin and vehicle were given to mice after *ip* administration of the combination of anaesthetic and analgesic (Narketan/Xylapan). Anesthetized animals were sacrificed 24 h after the last dose of MC, silymarin and vehicle. Previously, blood was collected from the orbital sinus and the serum was separated for determination of ALT activity. The livers were removed, washed with saline, blotted dry and divided into samples. Tissue specimens were frozen in liquid nitrogen and stored at -80 °C if not used for analysis immediately. Liver samples were used to assess the Cu/Zn SOD activity, protein content and for Western blot. Additionally, liver samples were preserved in a 4 % paraformaldehyde solution for histology and immunohistochemistry.

### Biochemical analysis

Serum levels of ALT were measured using a Bio-Tek EL808 Ultra Microplate Reader (BioTek Instruments, Winooski, VT, USA) according to manufacturer’s instructions.

Liver samples used for biochemical analysis were homogenized in 50 mM PBS (phosphate buffered saline, pH 7.4) using a Polytron homogenizer (Kinematica, Lucerne, Switzerland) and the supernatants were separated using Beckman L7-65 Ultracentrifuge (Beckman, Fullerton, USA) at 15,000 *g* for 20 min, at 4 °C. The supernatants were used for determination of Cu/Zn SOD activity, which was measured spectrophotometrically at 550 nm, based on the decrease in cytochrome c reduction by superoxide radicals generated in the xanthine/xanthine oxidase system, as described previously [18]. Cu/Zn SOD activity was expressed as units per mg of protein. The protein content in liver homogenates was estimated by Bradford’s method [19].

### Histopathological procedure

Liver tissues were placed in plastic cassettes and immersed in 4 % paraformaldehyde for 48 h. The fixed tissues were processed as described previously [20]. The degree of hepatocellular damage was evaluated by measuring the area of necrosis in liver sections stained with hematoxylin and eosin. For this purpose, we used light microscopy (Olympus BX51, Tokyo, Japan). The necrotic zones were manually selected and the percentage of necrotic area was determined using the Cell F v3.1 software, Olympus Soft Imaging Solutions (Münster, Germany).

### Immunohistochemical analysis

For immunohistochemistry, 4 μm thick deparaffinised liver tissue sections were used, as described earlier [20]. Briefly, deparaffinised liver slices were incubated overnight with the antibodies against NF-κB, Nrf2 and α-SMA. For antibody detection DAKO EnVision + System, Peroxidase/DAB kit was employed. The sections were then counterstained with hematoxylin, dehydrated using graded alcohols and xylene, and mounted with Entelan. The immunostaining intensity was analyzed by light microscopy (Olympus BX51, Tokyo, Japan). The Cell F v3.1 software, Olympus Soft Imaging Solutions (Münster, Germany), was used to quantify immunohistochemical staining across 10 high-power fields (400x).

### Western blot analysis

Western blot analysis with monoclonal antibodies against 4-HNE, TNF-α, HO-1 and TGF-β1 was performed as described by [20]. Briefly, liver samples were lysed in RIPA (radioimmunoprecipitation assay) buffer with added protease inhibitors. Volumes equivalent to 50 μg of proteins were transferred to 12 % polyacrylamide gel. After electrophoresis, gels were blotted onto polyvinylidene fluoride membrane. Membranes were blocked overnight by milk blocking reagent at 4 °C. The proteins were visualized by addition of the respective antibodies, followed by peroxidase-labeled goat anti-mouse F(ab’)2. The β-actin was used as a control of protein load. Membranes were washed with TBST buffer (Tris–HCl-buffered saline, 50 mM, pH 7.5, with 0.1 % Tween 20), incubated with Amersham ECL Prime (GE Healthcare, Uppsala, Sweden) and scanned (Kodak Image Station 440CF, Kodak, New Haven, CT, USA). The intensity of the bands was quantified using ImageJ software (NIH, Maryland, USA).

### Statistical analysis

Data were analyzed using StatSoft STATISTICA version 10.0 software by Kruskal-Wallis test and post hoc comparisons were carried out with Dunn’s multiple comparison test. Results of multiple comparisons tests were indicated by different letters. Means with letters in common are not significantly different from each other. Values in the text are means ± standard deviation (SD). Differences with *P* < 0.05 were considered to be statistically significant.

## Results

### Phenolic content of *M. croatica*

**Fig. 1:**
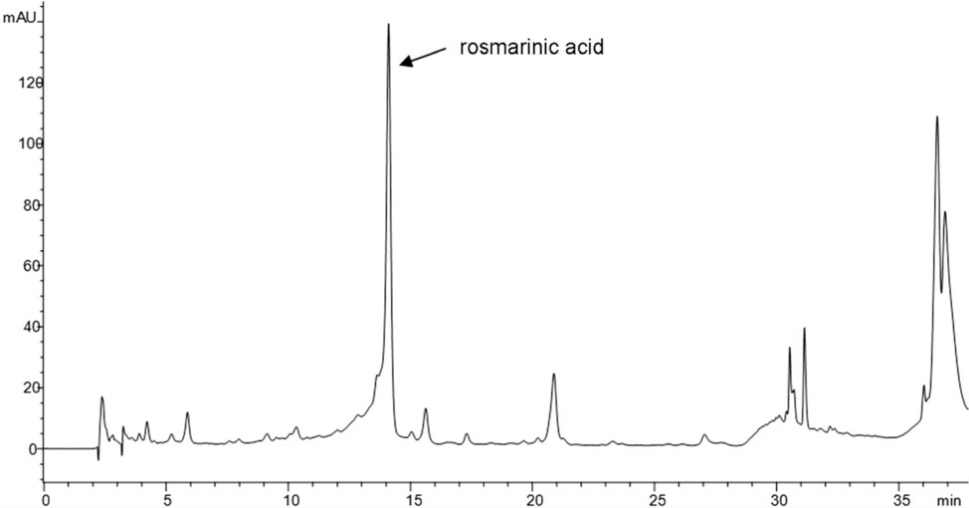
HPLC chromatogram of *Micromeria croatica* ethanolic extract recorded at 330 nm. The main compound was identified as rosmarinic acid (Rt = 14.1 min)

HPLC was employed to generate chromatographic profile of the *Micromeria croatica* ethanolic extract (MC) and to identify and quantify major constituents. As shown in Fig. 1, HPLC analysis revealed that rosmarinic acid (Rt = 14.10 min) was the main phenolic acid existed in the extract in amount of 9.95 mg/g extract. MC also contained 1.26 mg/g of chlorogenic acid (Rt = 4.22 min) and 0.47 mg/g of caffeic acid (Rt = 5.87 min), respectively.

### Liver weight and serum markers of liver damage

As can be seen from Table 1, relative liver weight of mice treated with the MC alone was similar to those of control group. CCl_4_-intoxication induced a significant increase in mice liver weight, which was significantly ameliorated by administration of MC in a dose-dependent manner. Serum ALT was significantly increased after CCl_4_ treatment (Table 1). Significant reduction of ALT activity was determined in the serum of MC400 groups, which was comparable to silymarin-treated animals.

**Table 1:** Relative liver weight and serum ALT and Cu/Zn SOD activities in the livers of CCl_4_-intoxicated mice treated with *Micrometria croatica* ethanolic extract (MC) and silymarin (S)

	Relative liver weight	ALT (U/L)	Cu/Zn SOD (U/mg protein)
Control	4.59 ± 0.62^a^	26.5 ± 1.8^a^	9.36 ± 0.63^a^
MC 400 mg/kg	4.75 ± 0.44^a^	29.4 ± 2.1^a^	9.12 ± 0.94^a^
CCl_4_	7.76 ± 0.87^b^	1447 ± 195^b^	5.22 ± 0.86^b^
CCl_4_ + MC 50 mg/kg	7.72 ± 0.46^b^	1406 ± 165^b^	5.71 ± 0.74^b^
CCl_4_ + MC 200 mg/kg	6.88 ± 0.32^b^	789 ± 107^b^	6.94 ± 0.37^b^
CCl_4_ + MC 400 mg/kg	5.75 ± 0.83^a^	293 ± 79^c^	8.17 ± 0.69^a^
CCl_4_ + S 400 mg/kg	5.74 ± 0.71^a^	256 ± 96^c^	8.29 ± 0.83^a^

Relative liver weight is expressed as g of liver weight/100 g of body weight

*ALT* alanine aminotransferase; *Cu/Zn SOD* superoxide dismutase

Each value represents the mean ± SD for 5 mice. Means sharing the same letter within row are not significantly different from each other (*P* < 0.05)

### Histopathological assessment of liver samples

**Fig. 2:**
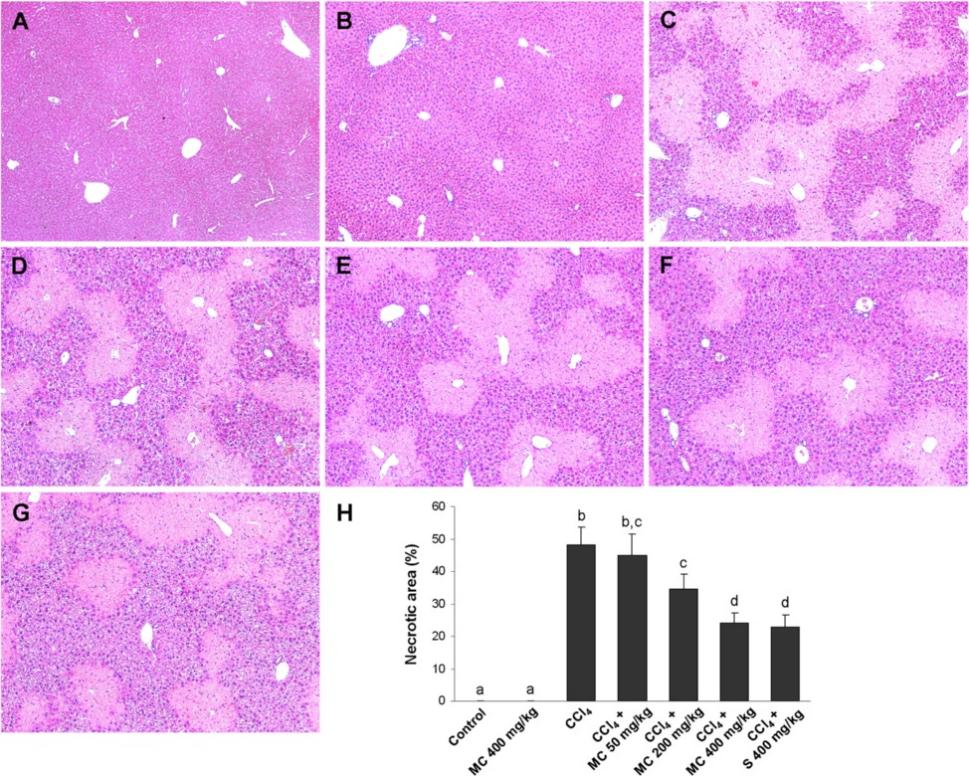
Liver histopathology. Control mice (**a**), mice receiving MC orally at 400 mg/kg (**b**), CCl_4_-intoxicated mice (**c**), and mice secondary treated with MC at doses of 50 mg/kg (**d**), 200 mg/kg (**e**) and 400 mg/kg (**f**) and silymarin at dose of 400 mg/kg (**g**). The necrotic area size measurement (**h**). Representative results from 5 similarly treated mice. Each value represents the mean ± SD (*n* = 5). Means sharing the same letter are not significantly different from each other (*P* < 0.05)

Histopathological analysis revealed that the livers of control mice (Fig. 2a) and mice treated with MC alone (Fig. 2b) had a normal histoarchitecture. CCl_4_ intoxication induced a severe centrilobular necrosis and loss of hepatic tissue, with mild inflammatory cell infiltration around the central vein region and sinusoidal spaces (Fig. 2c). In the livers of CCl_4_-intoxicated mice treated with MC at doses of 50 mg/kg (Fig. 2d), 200 mg/kg (Fig. 2e) and 400 mg/kg (Fig. 2f) an improvement in the morphology and reduction in both the size of necrotic areas and inflammatory infiltration was observed. The extent of hepatoprotection afforded by the extracts in the MC400 group against the CCl_4_-induced liver injury was similar to the reference compound silymarin (Fig. 2g and 2h).

### Antioxidant activity of *M. croatica*

**Fig. 3:**
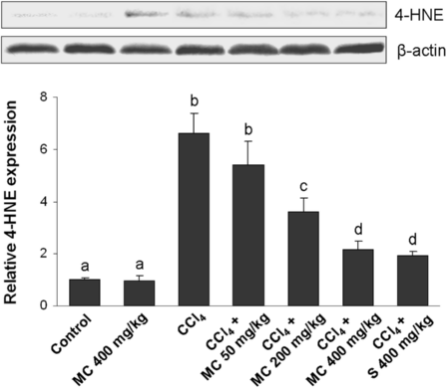
Western blot analysis of hepatic 4-hydroxynonenal (4-HNE) expression. Line 1: control mice, Line 2: mice receiving MC 400 mg/kg, Line 3: CCl_4_-intoxicated mice, Lines 4, 5 and 6: mice secondary treated by MC at doses of 50 mg/kg, 200 mg/kg and 400 mg/kg, respectively, Line 7: mice treated by silymarin at dose of 400 mg/kg. Each value represents the mean ± SD (*n* = 5). Means sharing the same letter are not significantly different from each other (*P* < 0.05)

**Fig. 4:**
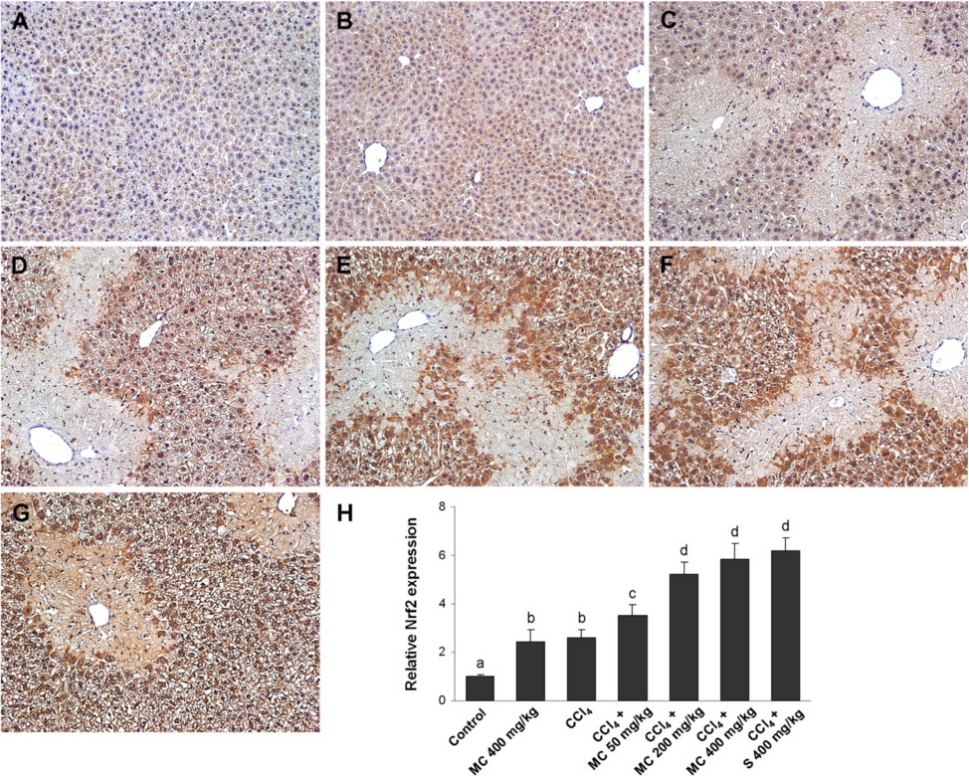
Hepatic NF-E2-related factor 2 (Nrf2) expression. Nrf2 immunostaining in control mice (**a**) and mice receiving MC 400 mg/kg (**b**). Nrf2 immunoreactivity in CCl_4_-intoxicated mice (**c**) and mice secondary treated by MC at doses of 50 mg/kg (**d**), 200 mg/kg (**e**) and 400 mg/kg (**f**) and silymarin at dose of 400 mg/kg (**g**). Original magnification 400x. Representative results from 5 similarly treated mice. Each value represents the mean ± SD (*n* = 5). Measurement of the Nrf2 immunostaining intensity (**h**). Means sharing the same letter are not significantly different from each other (*P* < 0.05)

Administration of CCl_4_ caused a significant decrease in Cu/Zn SOD activity (Table 1) and the increase in 4-HNE formation (Fig. 3) when compared to control group. Treatment with MC dose-dependently ameliorated changes in the hepatic Cu/Zn SOD activity and 4-HNE formation. At the dose of 400 mg/kg MC caused a similar increase in SOD activity and reduction of 4-HNE production as in the reference group. Further analysis of the antioxidant status in mice livers revealed low Nrf2 immunostaining in control mice (Fig. 4a). However, the increase in Nrf2 immunopositivity in the cytoplasm and the nuclei of hepatocytes and in Kupffer cells of mice treated with MC alone (Fig. 4b) as well as in mice intoxicated with CCl_4_ (Fig. 4c) was observed. Furthermore, a dose-dependent manner increase in Nrf2 immunostaining intensity was detected after administration of MC (Fig. 4d, e and f). High expression of Nrf2 was also found in the livers of silymarin-treated group (Fig. 4g), which was comparable to MC400 (Fig. 4h). Western blot analysis showed that HO-1 protein expression in the liver of control mice was negligible (Fig. 7). The expression of HO-1 significantly increased in the livers of CCl_4_-treated mice. Interestingly, administration of MC resulted in a dose-dependent increase in hepatic HO-1 protein expression. No statistically significant difference in HO-1 levels was observed between MC400 and silymarin groups.

### Anti-inflammatory activity of *M. croatica*

**Fig. 5:**
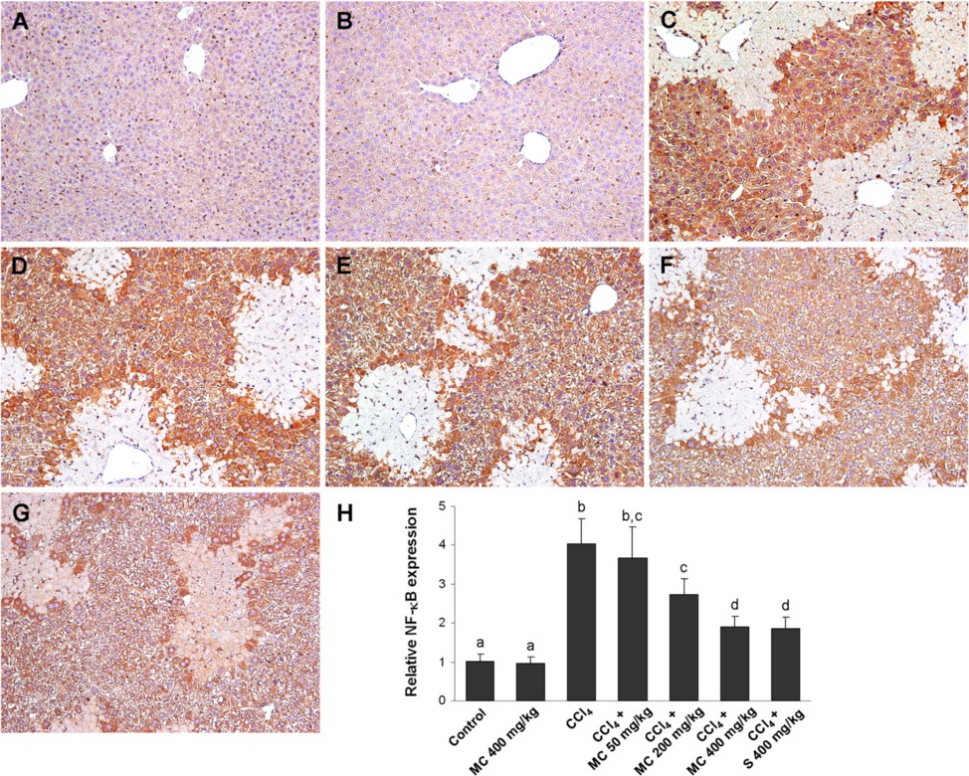
Nuclear factor-kappaB (NF-κB) p65 expression in the livers. NF-κB immunostaining in control mice (**a**) and mice receiving MC 400 mg/kg (**b**). NF-κB immunoreactivity in CCl_4_-intoxicated mice (**c**) and mice secondary treated by MC at doses of 50 mg/kg (**d**), 200 mg/kg (**e**) and 400 mg/kg (**f**) and silymarin at dose of 400 mg/kg (**g**). Original magnification 400x. Representative results from 5 similarly treated mice. Measurement of the NF-κB immunostaining intensity (**h**). Means sharing the same letter are not significantly different from each other (*P* < 0.05)

Immunohistochemical analysis of the NF-κB expression showed low expression of NF-κB p65 in the livers of control mice (Fig. 5a) and mice treated with MC alone (Fig. 5b), whereas strong NF-κB immunopositivity was found in the livers of CCl_4_-treated animals (Fig. 5c). Treatment with MC at 50, 200 or 400 mg/kg reduced NF-κB p65 immunopositivity in a dose dependent manner (Fig. 5d, e and f). Low expression of NF-κB was also found in the livers of silymarin-treated group (Fig. 5g), which was comparable to MC400 (Fig. 5h). Western blot analysis revealed that TNF-α protein expression in the livers of control and MC treated mice was negligible (Fig. 7). CCl_4_-intoxication significantly increased TNF-α expression, however, oral administration of MC resulted in a dose-dependent decrease in TNF-α production in mice livers. The hepatic expression of TNF-α in silymarin-treated mice did not show a statistically significant difference compared to MC400 group.

### Antifibrotic potential of *M. croatica*

**Fig. 6:**
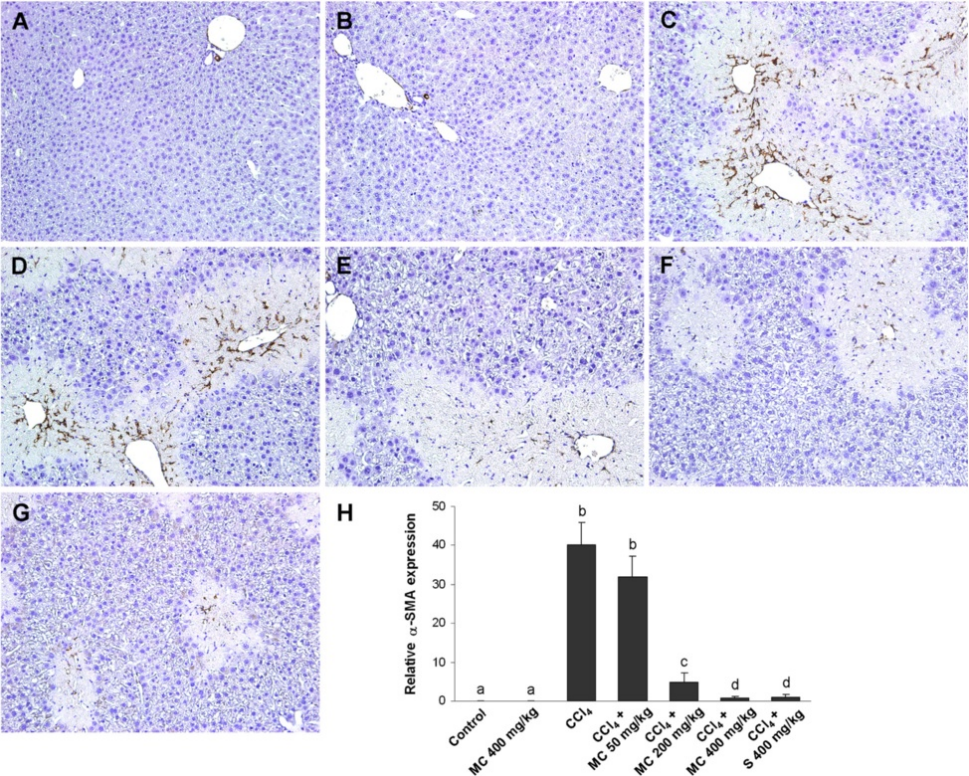
Hepatic alpha-smooth muscle actin (α-SMA) expression. α-SMA immunostaining in control mice (**a**) and mice receiving MC 400 mg/kg (**b**). α-SMA immunoreactivity in CCl_4_-intoxicated mice (**c**) and mice secondary treated by MC at doses of 50 mg/kg (**d**), 200 mg/kg (**e**) and 400 mg/kg (**f**) and silymarin at dose of 400 mg/kg (**g**). Original magnification 400x. Representative results from 5 similarly treated mice. Measurement of the α-SMA immunostaining intensity (**h**). Means sharing the same letter are not significantly different from each other (*P* < 0.05)

**Fig. 7:**
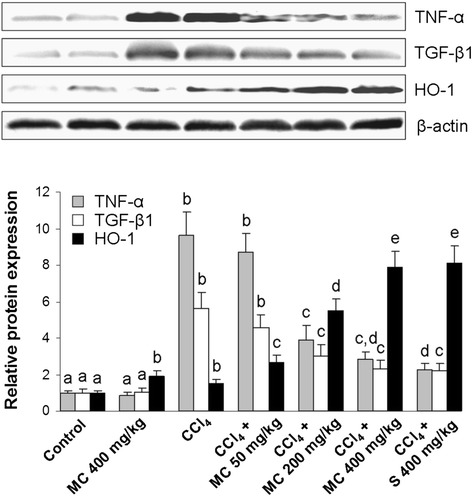
Western blot of hepatic tumor necrosis factor-alpha (TNF-α), transforming growth factor-beta 1 (TGF-β1) and heme oxygenase-1 (HO-1) expression. Line 1: control mice, Line 2: mice receiving MC 400 mg/kg, Line 3: CCl_4_-intoxicated mice, Lines 4, 5 and 6: mice secondary treated by MC at doses of 50 mg/kg, 200 mg/kg and 400 mg/kg, respectively, Line 7: mice treated by silymarin at dose of 400 mg/kg**.** Each value represents the mean ± SD (*n* = 5). Means sharing the same letter are not significantly different from each other (*P* < 0.05)

In the livers of control mice and MC group α-SMA immunoreactivity was not detected, except sporadically in the wall of blood vessels (Fig. 6a and b). In contrast, numerous α-SMA-positive cells emerged in the livers of CCl_4_-treated mice, indicating activation of hepatic stellate cells (HSC) (Fig. 6c). α-SMA immunopositivity was substantially reduced by oral administration of MC in a dose-dependent manner (Fig. 6d, e and f). The expression of α-SMA in the livers of MC400 group was similar to silymarin-treated mice (Fig. 6g and h). Changes in hepatic TGF-β1 expression coincided with changes in α-SMA immunopositivity, showing an increase in CCl_4_-intoxicated mice and a subsequent decrease by the MC treatment (Fig. 7). TGF-β1 and α-SMA expressions in the livers of MC400 group were comparable to mice receiving an equal dose of silymarin (Fig. 6h and 7).

## Discussion

In this study, we demonstrated that ethanolic extract of aerial parts of *Micromeria croatica* has preventive effect on CCl_4_-induced hepatic injury and fibrosis. Carbon tetrachloride (CCl_4_) is a potent hepatotoxin capable of causing experimental hepatic damage through the cytochrome P450-mediated activation to free radicals and reactive species, which in turn induce hepatocyte necrosis, inflammation and, subsequently, lead to liver fibrosis. Highly reactive radicals generated from CCl_4_, as trichloromethyl (^**·**^CCl_3_) and trichloromethyl peroxyl (^**·**^OOCCl_3_), affect the hepatocytes and cause structural and functional changes of their cellular membranes [21]. Our experiment showed that mice intoxicated with CCl_4_ developed a significant liver necrosis and oxidative stress, evidenced by a significant increase in the serum activity of ALT and impaired antioxidant status in the liver. The results of this study showed that the antioxidant constituents of MC acted against the damaging effects of free radicals produced by CCl_4_. The restoration towards normal level of serum ALT, the most sensitive biochemical marker of hepatocyte injury, indicated that MC preserved the structural integrity of hepatocellular membrane and protected the liver from the harmful effects of this hepatotoxin, which was additionally supported by the histological findings. Moreover, hepatoprotective effect of MC was accompanied by the amelioration of inflammation and an early onset of fibrogenesis in injured livers.

A number of recent studies suggested that the antioxidant activity against free radicals may be an important mechanism of the hepatoprotection [4, 5, 18, 22]. MC was found to possess significant *in vitro* antioxidant property, being capable of directly quenching free radicals resulting in the termination of the radical chain reaction, act as reducing agent, and chelate transition metals to suppress the initiation of radical formation [10]. Considerable antioxidant effects of MC, mainly attributed to the high level of phenolic compounds, especially rosmarinic acid, indicates that *M. croatica* could be useful in the prevention or amelioration of oxidative damage. In experimental animals, CCl_4_-mediated oxidative stress is associated with a lower antioxidant capacity of hepatic cells, characterized by a decreased functioning of the enzymatic and non-enzymatic defence mechanisms [23].

Acting as a first line of antioxidant defence*,* Cu/Zn SOD is a key enzyme in the cellular defence against the deleterious action of free radicals and other ROS, which in turn readily inactivate it. Considering that membrane lipids are the most susceptible to oxidative attack, the lipid peroxidation is recognized to be one of the major contributors to oxidative cell death and tissue injury. Increased steady-state level of the 4-hydroxynonenal (4-HNE), which is important end product of lipid peroxidation, is often taken as a specific marker of oxidative stress [24]. In the current study, SOD activity was brought to increase while 4-HNE decreased by the MC treatment of CCl_4_-intoxicated mice, evidently demonstrating its antioxidant effectiveness *in vivo*.

In order to clarify the mechanism of hepatoprotection, the effect of MC on activation of the Nrf2/HO-1 pathway was examined. Nrf2 is a key transcription factor which plays a central role in cellular defence against oxidative stress, inducing expression of a variety of cytoprotective and phase-2 detoxifying genes [25]. Heme oxygenase-1 (HO-1), one of the most important cytoprotective enzymes, has been shown to have a putative role in several different models of hepatic injury [26]. This inducible enzyme catalyzes the rate-limiting step of free heme degradation into free iron, carbon monoxide, and biliverdin, the last of which is subsequently catabolised into bilirubin, a potent endogenous antioxidant [27]. Furthermore, HO-1 functions as a suppressor of the proinflammatory TNF-α/NF-κB signalling pathway by diminishing intracellular ROS production [12]. Therefore, HO-1 is recognized as an important therapeutic target for pharmacological intervention of liver injury. The present study revealed MC-induced increase in Nrf2 and HO-1 expression in CCl_4_-intoxicated mice livers. High levels of these proteins were also determined in the livers of intoxicated mice treated with silymarin. These results are in agreement with other studies, which indicated that natural phenolics exert hepatoprotective effects via HO-1 induction [28, 29]. The induction of HO-1 via Nrf2 signalling by MC may provide an effective means for achieving cellular protection. Most recently, Park et al. [30] demonstrated that the induction of HO-1 was critical for anti-inflammatory effect of *Inula helenium* extract in LPS-activated RAW 264.7 macrophages.

The NF-κB pathway plays a central role in the inflammatory response. This pathway is activated upon appropriate cellular stimulation, most often by signals related to pathogens or stress [31]. Regulation and control of NF-κB activation can be a powerful therapeutic strategy for reducing tissue damage or other complications as a consequence of the release of inflammatory mediators. Several naturally occurring inhibitors of NF-κB, including caffeic acid, curcumin, resveratrol and silymarin, demonstrated antinecrotic, anticholestatic, antifibrotic and anticancer effects in the liver of experimental animals [32]. NF-κB activation, induced by oxidative stress, modulates hepatic damage by triggering cytotoxic cytokine production [33]. TNF-α is a proinflammatory cytokine that is rapidly produced in response to tissue injury and identified to be a key regulator of the inflammatory response. This pleiotropic cytokine stimulates the release of other cytokines from macrophages and induces the phagocyte oxidative metabolism as well as production of nitric oxide that can aggravate oxidative stress [34]. As we previously reported [18, 20, 35], natural antioxidants can significantly ameliorate proinflammatory response. In the current study, treatment with MC reduced NF-κB activation and TNF-α protein expression, suggesting the anti-inflammatory activity of MC.

Oxidative stress-related molecules may stimulate cellular events required for the development of liver fibrosis [36]. CCl_4_ is assumed to initiate free radical-mediated lipid peroxidation, leading to the accumulation of lipid-derived oxidation products that cause liver injury and excess collagen deposition in the liver, resulting in liver fibrosis [37]. Infiltration of inflammatory cells could contribute to hepatic oxidative stress and fibrogenesis. Ali et al. [38] showed that these cells, at least in part, belong to CD68+ macrophages, which have been suggested as important activators of hepatic HSCs [39]. Transforming growth factor-β1 (TGF-β1) is generally accepted as the main profibrotic factor. Our previous research pointed out that antioxidants of plant origin, such as delphinidin and oleuropein [20, 35] possess antifibrotic potential through down-regulation of TGF-β1 expression. In the present study, MC treatment markedly suppressed TGF-β1 production, which coincided with decreased α-SMA expression, an indicator of HSC inactivation, suggesting a potential to prevent an early onset of hepatic fibrogenesis. Rosmarinic acid, the most abundant polyphenolic constituent found in MC and a strong antifibrotic agent [18, 40], is very likely to contribute to the observed effect.

## Conclusions

The present study demonstrated for the first time the therapeutic properties of MC against liver injury. In the livers of chemically-intoxicated mice, the MC treatment was found to ameliorate oxidative stress, inflammation and fibrogenesis. Hepatoprotective and antifibrotic effects of MC were comparable to those of silymarin, a well known standard hepatoprotectant. These findings suggest that *M. croatica* could be useful in the treatment of acute liver injury and encourage further research to assess its value in modern phytotherapy.
